# Association between rheumatoid arthritis and periodontitis in an adult population – A cross sectional study

**DOI:** 10.4317/jced.57562

**Published:** 2021-10-01

**Authors:** Shailesh Varshney, Manish Sharma, Sanjeev Kapoor, M Siddharth

**Affiliations:** 1 Periodontist, MDS, Department of Periodontology, School of Dental Sciences, Greater Noida; 2 Post Graduate Student, Department of Periodontology , School of Dental Sciences, Greater Noida; 3 Rheumatologist, MD, DM, Maharaj Agrasen Hospital, Punjabi Bagh, Arthritis Unit, Department of Rheumatology, New Delhi

## Abstract

**Background:**

This investigation was aimed to analyse the existence of an association between rheumatoid arthritis and periodontitis among Indian subjects.

**Material and Methods:**

This observational study included a total of 110 individuals between 18-78 years of age, which were divided equally into RA (Rheumatoid Arthritis) and NRA (Non-Rheumatoid Arthritis) groups. General, Oral and a complete Periodontal examination included recording of Gingival Index (GI), Plaque index (PI), Pocket Probing Depth (PPD), Clinical attachment level (CAL) in a questionnaire form. Laboratory and rheumatologcal parameters like C-reactive protein (CRP), Erythrocyte sedimentation rate (ESR) and Disease Activity Score 28(DAS 28), Health Assessment Questionnaire–Disability Index (HAQ), Rheumatoid factor (RF) were also respectively estimated.

**Results:**

Prevalence of moderate or severe periodontitis was higher in RA than in NRA group. (41.8% vs 23.6%, *p*= 0.047). Periodontal structural damage represented by clinical attachment level was more in RA patients (2.89 mm v/s 2.54mm, *p*=0.261). Mean score of HAQ was co-related significantly in patients with CAL ≥ 2mm than with CAL < 2mm (0.69 v/s 0.455, *p*=0.0415). Through logistic regression analysis, periodontitis and CAL were related to RA with OR (Odds Ratio) of 2.1 and 2.89 respectively.

**Conclusions:**

Indian RA patients have higher odds for periodontitis and CAL may act as a risk indicator for RA.

** Key words:**Chronic periodontitis, rheumatoid arthritis, disease activity score 28, health assessment questionnaire, rheumatoid factor, c-reactive protein , erythrocyte sedimentation rate.

## Introduction

Periodontitis is a chronic inflammatory condition of bacterial etiology, which affects the supporting apparatus present around the teeth and is distinguished by derangement of the host immuno-inflammatory response, eventually leading to progressive destruction of soft and hard tissues of the periodontium (1).

On the other hand, the autoimmune disorder rheumatoid arthritis (RA) (2) dysregulates several systems and organs of the human body, leading to the destruction of both joint connective tissue including bone.

Moreover, these two chronic conditions exhibit a discrepancy in the release of pro and anti-inflammatory cytokines, which can be held accountable for the occurrence of tissue destruction. So in this respect, these two diseases present similarity in the destruction of bone (3), mediated by the release of inflammatory cytokines such as prostaglandin E2, Interleukin-1, Tumor necrosis factor-α etc (4).

Thus a two-way interrelationship between periodontitis and RA may exist in which periodontitis affects the pathogenesis of RA (5) and vice-versa (6).

Occurrences of bacteremia, the release of various types of inflammatory cytokines, bacterial antigens and immunoglobulins in the serum are some of the several pathways that have been proposed to denote periodontitis’s interference with the pathogenesis of RA (7). Serological studies conducted in humans (8) and mice (9) which estimated rheumatoid factor, support this perception. Higher titres of serum rheumatoid factor are associated with the activity, greater severity and less favourable outcome of RA (10). Additionally, periodontitis may have systemic consequences which may lead to aggravation of inflammatory mediator levels and recurrent intermittent bacteremias prevailing for an elongated period time (7).

RA may also impact the process of periodontitis through impairment of emotional and motor activity in individuals (11). The reduced or compromised motor activity makes it harder for these individuals to carry out and maintain a sufficient amount of oral hygiene. Intake of various antirheumatic drugs or the presence of secondary Sjögren syndrome in them might reduce their salivary flow and this could lead to an increase in the formation of supragingival plaque in these individuals (12). Psychological aberrations observed among RA subjects (13) were proposed as risk indicators for periodontitis.

Increased prevalence of periodontitis was reported among RA patients compared to systemically healthy control in cross-sectional studies (14) with odds Ratio (OR) ranging between 1.82 to 8.1. In contrast, there are cohort and case-control studies (15,16) which have shown no increase in either prevalence or incidence of periodontitis among RA patients.

Thus scientific investigations concerning the association between RA and chronic periodontitis are still inconclusive. The approaches employed among various investigations were as variable as their results and conclusions (17-19). Also there were no definitive results found, as the literature correlating the severity of periodontal disease and RA were limited or scant. Therefore, it requires further validation (20). Hence, this study was aimed to verify the hypothesis that whether subjects with rheumatoid arthritis are more likely to encounter severe periodontitis and to find out the most significant indicator of rheumatoid arthritis activity.

## Material and Methods

This was an observational study which had a cross-sectional design and was approved by the Institutional Ethics Committee, School of Medical Sciences and Research and Sharda Hospital, Sharda University.

A total of 110 Indian subjects participated in our study which was distributed equally into two separate groups.

1. RA Group – (Rheumatoid Arthritis Group )

2. NRA Group – (Non-Rheumatoid Arthritis Group)

The inclusion criteria for both the groups were: the volunteer of either gender; ≥ 18 years old; having ≥ 20 remaining teeth in their oral cavity; being willing to give informed consent and comply with all study protocols. The exclusion criteria included were the history of smoking/tobacco consumption; presence of systemic diseases or conditions that can modify periodontal disease like Diabetes Mellitus, Leukaemia, Hyperparathyroidism and Pregnancy; individuals who had taken any form of periodontal treatment and/or antibiotic therapy over a period of last six months.

Subjects in the RA group consisted of 55 patients taken from the Outpatient Arthritis Unit of Maharaja Agrasen Hospital, New Delhi and who had been diagnosed with RA by a rheumatologist based on the American College of Rheumatology and European League against Rheumatism (ACR/ EULAR) 2010 revised criteria (21). Specific inclusion criteria in this group of individuals were a diagnosis of RA for a period of beyond one year and the non-existence of any other autoimmune conditions.

The Non-Rheumatoid arthritis group was a convenience sample which consisted of 55 subjects selected by a process of randomisation from the Department of Oral Medicine & Radiology of the same dental college. The specific inclusion criterion for this category of individuals was the non-existence of RA or any other autoimmune conditions.

-Clinical Protocol and Data Procurement

All subjects filled a standardized Health questionnaire (general and oral) which included medical history, use of medication over a minimum period of six months, habits and oral hygiene procedures.

-Measurement of Periodontal parameters

Each of the 110 subjects from both the groups underwent an examination of their oral cavity which was performed by the same operator who was previously trained for recording all the periodontal parameters as per the need of the study and was also calibrated for measurement of Probing Pocket depth (PPD) and Clinical attachment level (CAL). CAL was measured as the distance from the Cemento-Enamel Junction (CEJ) to the base of the periodontal pocket whereas PPD was measured from the gingival margins to the base of the periodontal pocket.

Reproducibility of periodontal parameters was assessed by measuring the concordance between the data of two consecutive periodontal examinations performed at one week apart. A very good intra examiner agreement (90%) was obtained for the assessment.

Oral examination was also done to assess the number of teeth present, which was followed by the recording of:

1) Full mouth Gingival Index (GI) by Loe and Silness (1963).

2) Full mouth Plaque Index (PI) by Silness and Loe (1964).

3) Full mouth pocket probing depth measured at 6 sites per tooth excluding third molars - (mesio-mid-disto- lingual and mesio-mid disto-buccal) by graduated William’s periodontal probe.

4) Full mouth clinical attachment loss measured at 6 sites per tooth excluding third molars - (mesio-mid-distal lingual and mesio-mid disto-buccal) by graduated William’s periodontal probe.

Periodontitis was defined as the presence of four or more teeth with one site or more with PPD ≥ 4mm and CAL ≥ 2mm (22).

Periodontitis patients were further divided into slight (1-2 mm CAL), moderate (3-4 mm CAL) and severe (≥5mm CAL) periodontitis depending on the amount of CAL present (23).

Volunteers who did not have periodontitis were categorized as having gingivitis.

-Assessment of Rheumatological parameters

All patients in the Rheumatoid Arthritis group were evaluated for the amount of disability using Health Assessment Questionnaire–Disability Index (HAQ) (24) and disease activity using Disease Activity Score of 28 joints (DAS 28) (25).

Laboratory investigations were also done to determine the levels of Rheumatoid factor (RF), C-reactive protein (CRP) and Erythrocyte sedimentation rate (ESR) among all the volunteers of both the groups.

-Statistical analysis

The values thus recorded of various parameters were compiled and analysed by using the Statistical Package for the Social Sciences version 22.0 (SPSS Inc., Chicago, Illinois, USA). Descriptive statistics involved the calculation of percentages, means and standard deviations. Eventually, the data thus obtained was compared by Chi-square, Mann-Whitney-U and Student t-test. A probability value of < 0.05 was regarded as having a statistical significant difference. To determine the association between Periodontitis and Rheumatoid arthritis, various periodontal parameters were subjected to logistic regression analysis.

## Results

A total of 55 participants in RA and 55 participants in the NRA group took part in this study. These participants were between the age group of 18-78 years with the mean age of 42.2 years in the RA and 31.4 years in the NRA group (Table 1).

**Table 1:**
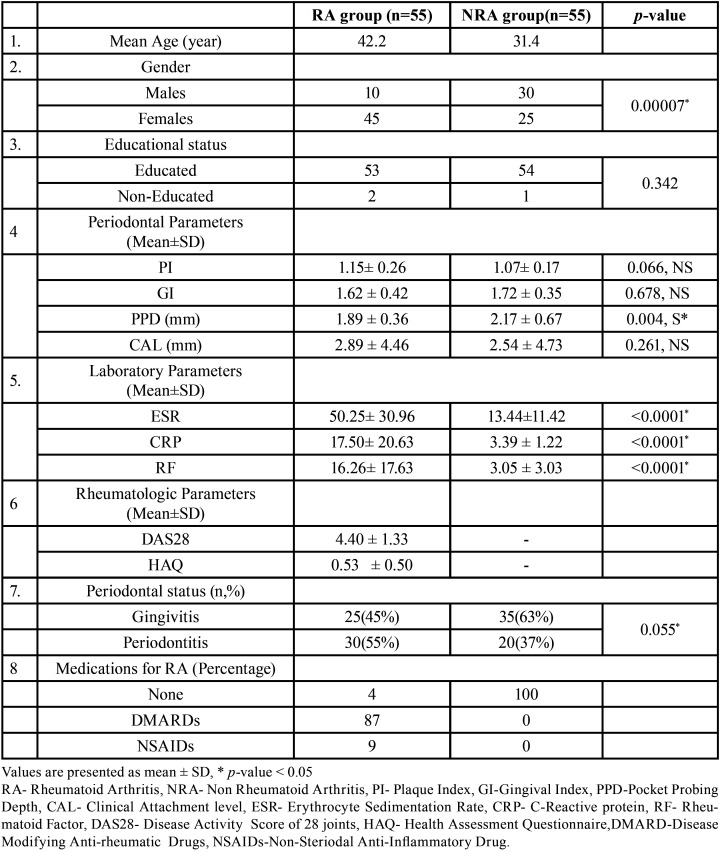
Demographic, Periodontal and Rheumatological characteristics of the Study Population.

There were 10 males compared to 45 females in the RA group and 30 males to 25 females in the NRA group (Table 1).

Moreover, there were more educated people than uneducated people in both groups (Table 1).

The prevalence of periodontitis was higher in the RA group (n=30,55%) when compared to the NRA group (n=20,37%) (Table 1).

The mean values of various periodontal parameters (PI, GI, PD and CAL) in subjects of both the groups are summarised in Table 1.

Though the amount of Gingival Inflammation and PD measured was more among subjects of NRA, mean plaque and CAL scores were higher in the RA group (Table 1). But still, these values did not reach to a level of statistically significant difference (Table 1).

Looking into the periodontal status of these subjects, statistically significant and a higher prevalence of moderate to severe periodontitis was seen among RA when compared to NRA group (*p*< 0.047) (Table 2).

**Table 2:**
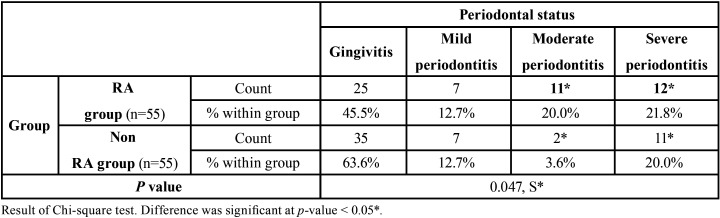
Distribution according to the different types of periodontitis of the study population.

For Laboratory parameters, values of RF, CRP and ESR were higher in the RA group compared to NRA group. Also, this difference observed among them was statistically significant (*p*<0.0001) (Table 1).

DAS 28 score in RA group was 4.40 indicating Moderate Disease activity whereas HAQ score was 0.53 indicating a mild to moderate disability (Table 1).

These Rheumatological characteristics, DAS 28 and HAQ were compared with periodontal parameter in subjects having CAL ≤ and > 2mm (Table 3). Mean HAQ score was found to be higher and statistically significant in individuals with CAL > 2mm (*p*< 0.0415, S) (Table 3).

**Table 3:**
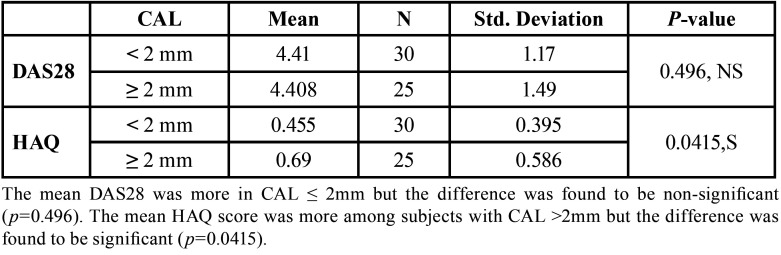
Rheumatologic characteristics (DAS28) & (HAQ) in subjects with RA with a mean CAL ≤ 2 or >2mm.

Multiple logistic regression analysis was also performed between various independent variables of periodontal status like Plaque Index, Gingival Index, Pocket depth, CAL and various categories of chronic periodontitis, among the subjects of both the groups, to identify the association between the two stated diseases (Table 4). The Odds ratio (OR) was determined. Odds of having Rheumatoid Arthritis was more than twice (OR= 2.1) (*p*= 0.05, statistically significant) in subjects with Periodontitis when compared to subjects not having periodontitis (Table 4). Among the various periodontal parameters compared in this analysis, subjects having CAL ≥ 2mm were found to be 2.89 times more prone (*p*=0.01, statistically significant) for developing Rheumatoid Arthritis when compared to subjects having CAL< 2mm (Table 4).

**Table 4:**
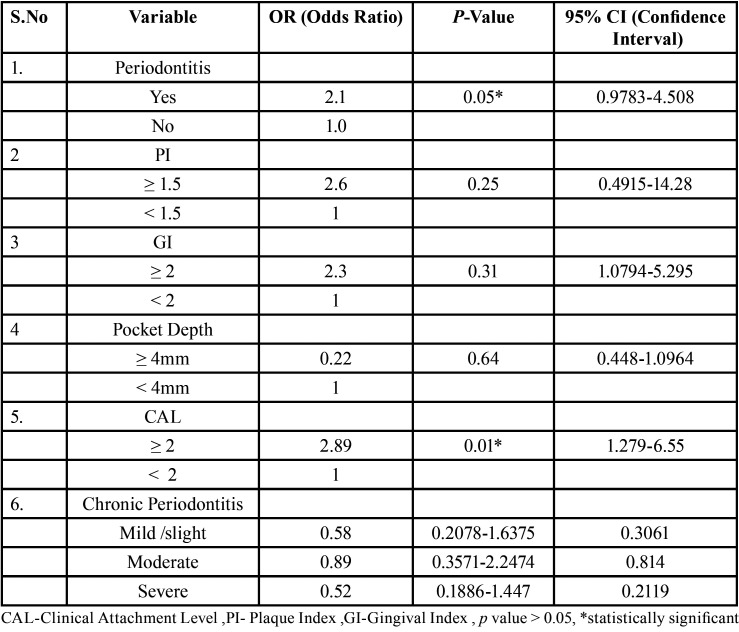
Result of Logistic regression analysis.

Other periodontal parameters evaluated did not find any significant relationship with Rheumatoid Arthritis (Table 4).

## Discussion

The present study compared the periodontal parameters in both RA and NRA patients. Additionally, it also correlated the Rheumatological parameters like DAS28 and HAQ with the CAL in the RA group (Table 3).

Higher Plaque index found in volunteers of the RA group (Table 1) could be due to lack of manual dexterity due to presence swollen and tender joints as indicated by higher DAS 28 and HAQ score (Table 1).

Although the RA group presented higher PI, but the gingival inflammation observed was less compared to NRA group (Table 1). This inverse variation may have been due to the sustained intake of anti-inflammatory and anti-rheumatic drugs over a long period of time, which is generally seen in RA patients. SAIDs (Steroidal Anti-Inflammatory Drugs), NSAIDs (Non-steriodal Anti-Inflammatory Drugs) and DMARDs (Disease Modifying Anti- Rheumatic Drugs) can modulate plaque-associated gingivitis (26).

Similarly one would expect a significant and higher amount of CAL in the RA group compared to NRA group of patients. But this was not the case in our study where CAL was only slightly higher and had an in-significant difference (*p*=0.261) (Table 1) in RA group compared to NRA group. This observation is analogous to the findings of the study done by Mirrielees *et al*. (27) in which clinical periodontal parameter recorded were lesser in RA and Healthy group compared to chronic periodontitis group.

Again the presence of in-significant and lesser amount of CAL in RA patient could be attributed to prolonged consumption of anti-rheumatic drugs as it has been suggested that management of Rheumatoid arthritis patients with disease modifying anti-rheumatic drugs (DMARD) leads to concealing of gingival inflammation and actual periodontal damage leading to improvement in periodontal status due to their host modulatory effects (26).

In our study, it was observed that higher prevalence of Periodontitis existed among Rheumatoid arthritis patients in comparison to NRA patients (Table 1) and also moderate to severe periodontitis was more prevalent in RA than NRA group (Table 2).

This explains the presence of a greater amount of CAL in RA patients when compared to NRA patients (Table 1) which is a better parameter than PD for measurement of periodontitis (28). In our study mean PD was found to be less in RA when compared to NRA group (Table 1).

 Our study also measured the laboratory parameters like ESR, CRP and RF which are indicators of the amount of systemic inflammation. These parameters were found to be greater in RA volunteers in comparison to NRA volunteers and were statistically significantly different (*p*<0.0001) (Table 1).

The above mentioned observations were in accordance with findings of other studies done to link the association of RA and periodontitis and its severity (5,19) This presence of a higher prevalence of periodontitis found in RA group of our study indicates that there exists a possible link between the manifestations of these two chronic inflammatory conditions.

There are noTable resemblances in the pathogenesis of these two chronic diseases at both molecular as well as the cellular levels. In both these diseases, cytokines are believed to play a crucial part in causing periodontal and joint tissue breakdown. The most important cytokines among them include TNF-α (Tumour Necrosis Factor-α) and IL-1 (Interleukin-1). Both of these are probably released by macrophages that are stimulated by T-cells. TNF-α and IL-1, then, in turn, stimulates synovial and periodontal ligament cells proliferation and thus produces a variety of inflammatory mediators and Matrix Metallo Proteinases (MMPs), which ultimately leads to cartilage breakdown of joints and alveolar bone destruction of the periodontium. Thus, a series of events are set up, that leads to sequential damage (29). Most of these relationships can also be elucidated in part by the enormous generation of pro-inflammatory cytokines and other inflammatory mediators among which IL-1, TNF-α and prostaglandin E2 (PGE2) appear to predominate (30).

The above mentioned link between RA and periodontitis could be associated when a periodontal parameter shows any link with a Rheumatologic parameter. For this, in our study values of CAL were compared with DAS 28 and HAQ scores.

Mean HAQ score was found to be more in patients with CAL ≥ 2mm when compared to CAL< 2mm (Table 3) and this difference was statistically significant (*p*=0.0415).

Though not many studies in the literature have compared clinical periodontal parameter with a clinical rheumatological parameter but this result could be comparable with the findings of the study conducted by Ribeiro *et al*. (6) in which HAQ analyses showed diminution in the degree of disability in a group of RA patients who had undergone periodontal treatment.

Thus this study provides some positive indicators correlating Periodontitis and Rheumatoid arthritis. These include.

1. Presence of more CAL in the RA group. 2.89 mm in the RA group followed by 2.54 mm in NRA group.

2. Moderate and severe periodontitis was significantly more prevalent in RA than the NRA.

3. Mean score of HAQ disability index significantly correlated with an increase in CAL level.

4. Odds for having rheumatoid was found to be higher in patients with periodontitis and CAL ≥ 2mm.

But these results have to be interpreted with caution as our study did have few limitations too, such as:

1. Cross-sectional nature of our study which complicates the drawing of causal inferences.

2. Smaller sample size, which may restrict the accuracy of the risk estimates.

3. Randomly selected subjects leading to significantly inconsistent demographic data such as age and sex.

## Conclusions

Role of periodontal infection on various systemic diseases or conditions has still been one of the many critical issues in the field of periodontal research. In most of these researches, it has been only implicated as a risk factor. Looking into our study, there are some positive outcomes relating periodontitis and rheumatoid arthritis and thus it could be designated as a contributing factor. Still prospective and interventional studies with a larger group of volunteers are warranted to validate our conclusions and endorse the hypothesis. Also for future planning, awareness regarding implications of poor oral hygiene, oral prophylaxis and early dental care should be reinforced in RA patients. This may have an excellent effect on their general health status.
